# Removing reference mapping biases using limited or no genotype data identifies allelic differences in protein binding at disease-associated loci

**DOI:** 10.1186/s12920-015-0117-x

**Published:** 2015-07-26

**Authors:** Martin L. Buchkovich, Karl Eklund, Qing Duan, Yun Li, Karen L. Mohlke, Terrence S. Furey

**Affiliations:** Department of Genetics, University of North Carolina, Chapel Hill, NC 27599 USA; Department of Biostatistics, University of North Carolina, Chapel Hill, NC 27599 USA; Department of Computer Science, University of North Carolina, Chapel Hill, NC 27599 USA; Department of Biology, University of North Carolina, Chapel Hill, NC 27599 USA

**Keywords:** Allelic imbalance, Genome mapping bias, Transcription factor binding, CREB1, Inflammatory bowel disease, Alleles, GWAS, ChIP-seq

## Abstract

**Background:**

Genetic variation can alter transcriptional regulatory activity contributing to variation in complex traits and risk of disease, but identifying individual variants that affect regulatory activity has been challenging. Quantitative sequence-based experiments such as ChIP-seq and DNase-seq can detect sites of allelic imbalance where alleles contribute disproportionately to the overall signal suggesting allelic differences in regulatory activity.

**Methods:**

We created an allelic imbalance detection pipeline, AA-ALIGNER, to remove reference mapping biases influencing allelic imbalance detection and evaluate accuracy of allelic imbalance predictions in the absence of complete genotype data. Using the sequence aligner, GSNAP, and varying amounts of genotype information to remove mapping biases we investigated the accuracy of allelic imbalance detection (binomial test) in CREB1 ChIP-seq reads from the GM12878 cell line. Additionally we thoroughly evaluated the influence of experimental and analytical parameters on imbalance detection.

**Results:**

Compared to imbalances identified using complete genotypes, using imputed partial sample genotypes, AA-ALIGNER detected >95 % of imbalances with >90 % accuracy. AA-ALIGNER performed nearly as well using common variants when genotypes were unknown. In contrast, predicting additional heterozygous sites and imbalances using the sequence data led to >50 % false positive rates. We evaluated effects of experimental data characteristics and key analytical parameter settings on imbalance detection. Overall, total base coverage and signal dispersion across the genome most affected our ability to detect imbalances, while parameters such as imbalance significance, imputation quality thresholds, and alignment mismatches had little effect. To assess the biological relevance of imbalance predictions, we used electrophoretic mobility shift assays to functionally test for predicted allelic differences in CREB1 binding in the GM12878 lymphoblast cell line. Six of nine tested variants exhibited allelic differences in binding. Two of these variants, rs2382818 and rs713875, are located within inflammatory bowel disease-associated loci.

**Conclusions:**

AA-ALIGNER accurately detects allelic imbalance in quantitative sequence data using partial genotypes or common variants filling a critical methodological gap in these analyses, as full genotypes are rarely available. Importantly, we demonstrate how experimental and analytical features impact imbalance detection providing guidance for similar future studies.

**Electronic supplementary material:**

The online version of this article (doi:10.1186/s12920-015-0117-x) contains supplementary material, which is available to authorized users.

## Background

Genetic studies of complex traits and diseases have been increasing their focus on the contribution of gene transcriptional regulation. The majority of complex trait-associated variants are in non-coding regions [1], suggesting many contribute by altering regulatory activity. Variants can alter transcription factor binding affinity, subsequently affecting transcription levels of target genes [1]. For example, the T allele of rs12740374 increases C/EBPa binding and transcription of *SORT1*, a gene influencing LDL cholesterol level [2]. Identifying precisely which genetic variants are responsible for changing regulatory activity can be difficult.

Quantitative short-read sequence data generated from experiments such as ChIP-seq [3], DNase-seq [4], FAIRE-seq [5], and ATAC-seq [6] broadly identify genomic regions that regulate gene transcription. Sequence information from these experiments can be used to detect allele-specific activity in samples where heterozygous variants are present in or near a regulatory element. For example, an uneven distribution in the number of reads containing each allele at a heterozygous site, referred to as allelic imbalance, provides evidence for differential regulatory activity due to genetic variation. Previous studies have also used quantitative short-read data to correlate genetic variation in regulatory regions with nearby gene expression [7, 8] and to show the heritability of allelic regulatory effects [8–12].

Quantitative sequence data have been generated in hundreds of cell types and tissues by the ENCODE (Encyclopedia of DNA elements) Consortium [13] and Roadmap Epigenomics Project [14]. Offering a valuable source of genetic regulatory information. Exploration of allelic imbalance in this data is hindered by a lack of complete genotype information for individuals from which these data are derived, and the well-established alignment bias that arises when both alleles at a heterozygous site are not considered during alignment to a reference genome. Sequence reads containing the allele not represented in the reference genome are penalized as an additional mismatch compared to reads containing the reference allele [15], and are less likely to map to the correct genomic location (Additional file 1: Figure S1). This can result in false detection of allelic imbalance favoring the reference allele, or failure to detect imbalance favoring the non-reference allele. Several methods for removing this alignment bias have been proposed, including masking known variants in the reference genome [15], aligning reads to two haplotype reference genomes [16–21], using known variants with allele-aware aligners [7, 8, 22] or creating an extended reference genome that included alternate alleles [23]. For these methods, full genotype information leads to the best results, but this data is rarely available. The performance of these methods using limited or no sample genotype data, compared to full genotype information has not been thoroughly investigated.

To evaluate detection of altered regulatory activity due to genetic variation in quantitative sequence data using full, limited or no genotype information, we created a computational analysis pipeline, called AA-ALIGNER (Allele-Aware ALignments for the Investigation of GeNetic Effects on Regulation). AA-ALIGNER strategically incorporates existing, publicly available tools to accurately annotate regions containing heterozygous variants given varying levels of genotype information, including no genotypes. To remove alignment biases at heterozygous variants, AA-ALIGNER uses the allele-aware aligner GSNAP [24] which has been previously shown to remove mapping biases using complete genotype information [22]. AA-ALIGNER also attempts to correct other biases that can influence imbalance detection, such as incorrect heterozygous site annotations in reference genome sequences and incorrectly detected imbalances due to differences in mappability between reads containing each of the alleles or due to PCR duplications introduced during sequencing [25].

We demonstrate that GSNAP also removes mapping biases using partial genotype data or common variants allowing for accurate identification of allelic imbalances. Using AA-ALIGNER, we determined the effect of experimental and analytical variables such as sequence read length, sequencing depth, number of mismatches allowed during alignment, and imputation quality thresholds on accurate allelic imbalance detection. Our analyses used data from one DNase-seq and thirteen ChIP-seq experiments generated in the GM12878 lymphoblastoid cell line, for which both complete, sequencing-based genotype and partial, array-based genotype information is available. We experimentally detected differential protein binding at six of nine tested imbalance predictions from AA-ALIGNER for CREB1 (Cyclic-AMP Responsive Element Binding protein 1) binding in GM12878 ChIP-seq data, including imbalances at two disease-associated loci. Overall, our results provide important empirical data that can be used to guide the design of and interpretation of similar studies using AA-ALIGNER to accurately annotate heterozygous sites and detect genetically-driven changes in regulatory element activity.

## Methods

### Genotype data

Genomic sequencing-based variants calls for GM12878 were generated by the Broad Institute. Illumina Human-1MDuo BeadChip array genotype data generated by the HusdonAlpha Institute of Biotechnology for GM12878 and 52 other ENCODE samples were obtained from the UCSC genome browser [26]. Autosomal genotypes for all 53 samples were imputed using MaCH-Admix [27] with default parameter settings and the reference panel from the 1000 Genomes Project Phase I version 3 (2012-03-14 release). Chromosome X genotype data in the 53 samples were pre-phased using MaCH [28] with options --states 500 and --rounds 400 and then imputed using minimac [29] with options --state 10 and --rounds 10. Post-imputation filtering of variants according to Rsq was performed as previously reported [30].

Common alleles (MAF > 0.05) used to derive the initial custom reference genome were based on 1000 Genomes Phase I version 3 EUR population [31].

### Custom reference creation

The initial European-specific reference genome was created by replacing alleles in the hg19 reference sequence with the major allele for all common variants (MAF > .05) from the 1000 Genomes EUR population. The GM12878 custom reference was created by further modifying this initial custom reference by replacing non-reference homozygous variants with the new allele, based on information from either the full genotype or partial genotype.

### Quantitative sequence data processing

Sequence fastq files (Additional file 2: Table S4) were downloaded from the UCSC Genome browser ENCODE Project [26]. Sequences from each replicate were filtered with fastx_trimmer using options ‘-f 1 -l 50 –Q 33’ and fastq_quality_filter using options ‘-Q 33 –p 90 –q 20 –I *N*’ where *N* is the length of the reads in that dataset.

Standard GSNAP alignments were performed using the following options: −-sampling = 1, −-terminal-threshold = 10, −n 1, −-query-unk-mismatch = 1, −-genome-unk-mismatch = 1, −-trim-mismatch-score = 0, −t 7, and -A sam. The k-mer size parameter was set based on read length: _−_k = 15 (50 bp); −k = 11 (35 bp); −k = 10 (20 bp) with –-basesize set to k-mer size. As we increased the number of mismatches allowed during alignment to *m*, we changed the option –m to *m* and –i to *m* + 1 to disallow indels during alignment. The directory containing the GSNAP reference genome was specified with –D the genome name with –d. Alternate alleles at variant sites based on partial genotype information or common variants were included in alignments with the –v option. BWA alignments were performed using the bwa aln command with options –n 1, −o 0, and –e 0 and bwa samse with option –n 4. When doing a second alignment, the customized reference was updated, if necessary, to contain one of the alleles at predicted heterozygous sites from the first alignment, sequences were aligned, and the alignments were filtered as before.

Reads aligned to more than one genomic location or overlapping the ENCODE blacklist regions [26] were filtered. Potential PCR artifacts were removed using MarkDuplicates (Picard suite) with options REMOVE_DUPLICATES = TRUE, VALIDATION_STRINGENCY = LENIENT, USE_THREADING = TRUE.

To investigate the effects of reference mapping biases on peak calling, peaks were called using SPP within an Irreproducible Discovery Rate (IDR) analysis [32] as outlined by the ENCODE Consortium [33, 54]. Overlaps were determined between the 10,000 peaks with the strongest signal and heterozygous sites identified by genomic sequencing (complete genotypes).

### Identifying allelic imbalance

Only sequence bases with a Phred33 base quality score greater than 30 were considered for predicting heterozygous sites or allelic imbalances. To account for mappability differences in alignments based on which of the two alleles was present, the heterozygous base in each sequence read was changed to the alternate allele and re-aligned to the genome. Only reads aligning uniquely regardless of the allele present were used to detect allelic imbalance. Significance was assessed with a binomial probability, b*(*a_1*;*_*n, 0.5),* where a_1_ represents the number of reads containing allele1 and *n* is the total number of reads at the heterozygous site and an uncorrected *p*-value threshold of 0.01. To calculate beta-binomial *p*-values, we first estimated parameter α of the beta distribution using reference allele proportions across all sites. A Z-statistic for each tested site was calculated the following equation:

$$ \frac{\widehat{\mathrm{P}}-0.5}{\sqrt{\frac{2\alpha +N}{4N\left(2\alpha +1\right)}}}, $$

where $\widehat{\mathrm{P}}$ is the proportion of reads containing the reference allele and N is the total number of reads at the site.

### Electrophoretic mobility shift assays

For each heterozygous variant examined, two sets of complementary 21-mer, biotin-labeled oligonucleotides centered on the CREB1 motif and containing one allele were synthesized by Integrated DNA Technologies. Each set was annealed to create two double-stranded probes for each variant (Additional file 2: Table S5). EMSAs were performed according to the protocol included with the LightShift Chemiluminescent EMSA Kit (Thermo Scientific). Briefly, each reaction containing 1x binding buffer, 1 μg poly(dIdC), and 200 ng of purified CREB1 protein (CreativeBiomart CREB1-26H) was incubated for 15 min before adding biotin-labeled probes in a total reaction volume of 20 μl and incubating for another 25 min. Reactions were electrophoresed on 6 % DNA retardation gels (Life Technologies) in 0.5X TBE buffer (Lonza), transferred to nylon membranes (Thermo Scientific), UV cross-linked and detected with chemilluminescence (Thermo Scientific).

### Availability of supporting data

The AA-ALIGNER pipeline package is available online [34].

## Results

### Overview of AA-ALIGNER

**Fig. 1:**
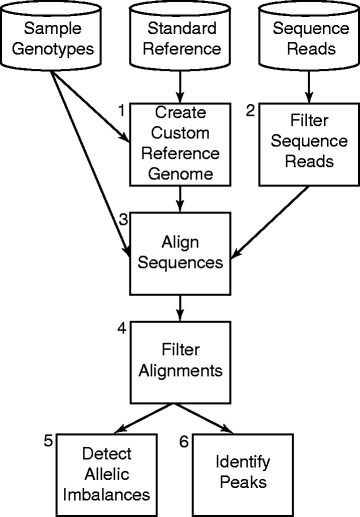
Overview of AA-ALIGNER. Sample genotypes or common variants are used to create a custom reference genome (1). Sequence reads are filtered to remove low quality reads (2) and aligned to the custom reference using GSNAP including alternate alleles (3). Alignments are filtered further to increase alignment quality (4) and used to detect sites of allelic imbalance (5, binomial test) and identify peaks (6). Allelic imbalance is tested at heterozygous sites included in the customized reference genome and at predicted heterozygous sites, identified based on a minimum number of mapped reads containing each of two alleles. If desired, predicted heterozygous sites can be used to update the custom reference and be included in a second alignment repeating steps 3–6

The AA-ALIGNER pipeline is designed to maximize short-read sequence alignment accuracy at sites of DNA variation regardless of genotype availability. These alignments can be used to identify potential sites of regulatory activity, indicated by an enrichment of aligned reads and referred to as peaks, and of allelic imbalance at these sites (Fig. 1). We first construct a sample-specific custom reference genome in a two-step process. To increase the likelihood that the allele in our starting reference genome matches the genotype of any sample, alleles of common variants in the standard reference are modified as needed to the most common allele from a particular population, such as the 1000 Genomes European samples [35]. In a second step, all available genotype information from the sequenced sample is used to further customize this reference sequence such that: (i) at homozygous variants, the sample allele is present; and (ii) at heterozygous sites, one of the two sample alleles is present. Alternate alleles at heterozygous sites are recorded in a separate file during this process. When no genotype information is available, this alternate allele file contains all common minor alleles (MAF > 0.05) for the selected population.

Next, we filter sequence reads to remove low quality sequences and align them to the custom reference genome using GSNAP [24], an allele-aware aligner. GSNAP takes as input the file containing reference and non-reference alternate alleles to equally consider alignments to both alleles. After alignment, we filter (i) sequences aligned to more than one genomic location; (ii) sequences aligned to regions underrepresented in the reference sequence (ENCODE blacklisted regions); and (iii) duplicate reads to correct for PCR artifacts. These final alignments are used to identify peaks and sites of allelic imbalance.

When testing for imbalances, AA-ALIGNER includes predicted heterozygous sites not included in the initial custom reference during sequence alignment. New heterozygous sites are predicted based on having a minimum number of reads containing each of two alleles. In addition, a minimum read threshold per allele can be applied to all heterozygous sites during imbalance detection to guard against incorrectly annotated heterozygous sites. While predicted heterozygous sites are not included in the initial reference genome customization (Fig. 1, Box 1) or sequence alignment steps (Fig. 1, Box 3), they can be added in a second round of reference customization and alignment if desired.

AA-ALIGNER is designed to correct for multiple sources of bias in the data whenever possible. Increasing the minimum read threshold required to test for an imbalance can guard against incorrect heterozygous site identification. Mappability biases, where reads containing one allele map uniquely while reads containing the other allele map to multiple locations and are filtered, may result in an artificial imbalance. AA-ALIGNER only considers reads that map uniquely to the same position in the genome regardless of the allele present. Post alignment filtering of duplicate reads corrects for biases that can arise from PCR duplication during library preparation.

AA-ALIGNER allows key parameters to be specified that influence sequence alignment and post-alignment steps, such as imbalance detection. The minimum read threshold for each allele is one of these parameters. In addition, allowed mismatches can be restricted to predicted heterozygous sites to increase confidence in evidence for multiple alleles. By default, significance of allelic imbalances is determined using a standard binomial test, but the AA-ALIGNER pipeline can be easily modified to incorporate alternative statistical methods of detecting imbalance. Peaks are determined here using SPP [36]. Additional details for individual steps can be found in the Methods. Unless otherwise indicated, the following results are based on alignments allowing for one mismatch, with a minimum of five reads required for each allele, and a nominal binomial *p*-value threshold of 0.01 for allelic imbalance detection. Each of these parameters is evaluated in detail in the following sections.

### Using GSNAP removes alignment biases at heterozygous sites

We first evaluated the ability of GSNAP to overcome the reference alignment bias. We used 50 base pair (bp) CREB1 ChIP-seq reads generated in the GM12878 lymphoblastoid cell line by the HudsonAlpha Institute of Biotechnology as part of the ENCODE project. We created a custom GM12878 reference sequence based on a complete set of genotypes generated by the Broad Institute [37], and we created a GSNAP input file with non-reference alleles for each heterozygous site. To examine whether both alleles at heterozygous sites were equally considered during alignments, we also created a “complement” reference sequence by swapping the allele at each heterozygous site in the initial custom reference with the alternate allele from the input file. We compared sequence alignments to these two reference sequences using three metrics: reads mapped to heterozygous sites; sequence enrichment peaks called at heterozygous sites; and sites of allelic imbalance (Table 1). Only 120 of the 33.6 million (0.0003 %) reads were aligned differently between the two alignments. Manual inspection indicated that these discrepancies were due to GSNAP failing to remove alignment bias when aligning sequences to regions containing more than 5 and as many as 16 heterozygous sites. These 120 differences did not affect the number of peaks or the predicted sites of allelic imbalances identified (Table 1). These data demonstrate that using GSNAP, AA-ALIGNER overcomes the alignment bias.

**Table 1:** Allele-aware alignments with complete genotypes (GSNAP) vs no genotype information (BWA)

	GSNAP			BWA		
	Standard	Complement^a^	Difference^b^	Standard	Complement^a^	Difference^b^
**Reads mapped uniquely**	33,599,679	33,599,721	120	33,543,808	33,547,947	344,942
**Reads at heterozygous sites**	1,295,901	1,295,914	120	1,197,696	1,186,891	344,942
Reference allele	675,394	620,517	-	677,697	640,978	-
Non-reference allele	620,507	675,397	-	519,999	545,913	-
**Peaks at heterozygous sites** ^**c**^	1618	1618	0	1593	1614	87
**Allelic imbalance sites identified** ^**d**^	200	200	0	151	147	56
Reference allele	108	92	-	91	82	-
Non-reference allele	92	108	-	60	65	-

^a^Alignment reference contained the non-reference allele of heterozygous sites used to create the standard reference ^b^Differs in mapping or detection between alignments to standard and complement references ^c^Out of 10,000 peaks with strongest signal ^d^binomial *p*-value < .01

To quantify the importance of removing the alignment bias, we used the same metrics to compare allele-aware and non-allele-aware alignments using the same reference sequences. We used BWA for non-allele-aware alignments with the same alignment parameters as GSNAP. By considering alternate alleles, GSNAP (1.3 M reads) aligned 8 % more reads to heterozygous sites than BWA (1.2 M reads; Table 1). As expected, GSNAP aligned a larger percentage of reads containing the non-reference allele compared to BWA (48 % to 43 %), more closely reflecting the expectation that each allele should be present in equal numbers of reads. Additionally, we aligned sequence reads to the complement reference using BWA. In contrast to GSNAP, we found that BWA aligned 344 K (1.0 %) reads differently to the complement and reference genomes. Greater than 54 % of reads mapped to the reference allele at heterozygous sites in both BWA alignments (Table 1), demonstrating the effect of alignment bias on non-allele-aware alignments.

We examined, separately, the effect of biased alignments at heterozygous sites on peak and allelic imbalance detection. Among the top 10,000 peaks with the greatest signal enrichment for each alignment method, using GSNAP identified 1.6 % more peaks overlapping a heterozygous site than BWA and predicted 32 % more allelic imbalances. Further, 54 % of GSNAP-identified imbalances were enriched for the reference allele compared to 60 % of BWA-identified imbalance sites (Table 1). Additionally, the reference allele was enriched in 82 % (23/28) of imbalances only detected when using BWA, compared to 49 % (39/79) of imbalances unique to GSNAP alignments. The majority of BWA imbalances favored the reference allele in both the standard reference and the complement reference, demonstrating the presence of significant alignment bias. Together, these results demonstrate that alignment biases negatively impact accurate sequence alignment, peak calling and allelic imbalance identification.

### AA-ALIGNER identifies sites of allelic imbalance using partial genotypes or common variant information

Complete genotypes are not available for most samples. Therefore, we evaluated how well AA-ALIGNER reproduced allelic imbalance annotations using incomplete genotype information. We separately aligned the same 50 bp CREB1 ChIP-seq reads to custom GM12878 reference genomes derived using (i) partial genotypes determined using the Human1M-Duo BeadChip array and imputed using MachAdmix [27]; and (ii) 1000 Genomes common variants (EUR, MAF > .05) to model the case of no available genotype information. Using allelic imbalances identified with complete genotype information to define true positive (TP), false positive (FP), and false negative (FN) sites, we calculated sensitivity (TP/TP + FN) and precision (TP/FP + TP), or positive predictive value.

Similar numbers of imbalances were identified using all three levels of genotype information (Table 2, Additional file 2: Tables S6–S8). Interestingly, we found that when simply including common variant alleles (no available genotypes), we detected imbalances with similar sensitivity (>73 %) and precision (>75 %) as with partial genotype information (Table 2). Including alleles of common variants with GSNAP significantly improved alignment performance compared to BWA with no variant information (Additional file 2: Table S2), even though neither alignment includes any information about the sample’s genotype. This improvement results from sites where including both alleles during alignment allowed for the imbalance to be detected. Of the 200 sites of imbalance detected using complete genotypes, 125 were present in the partial genotypes and 141 were common variants. Considering only these 125 and 141 sites, we find that sensitivity is 97 % and 94 % with 90 % and 82.5 % precision, respectively. In stark contrast, sensitivity of detection is 33 % (partial) and 34 % (common) with 45 % and 47 % precision at other predicted heterozygous sites, defined as sites with 5 or more reads containing each allele.

**Table 2:** Allelic imbalance detection accuracy in alignments using partial or no genotypes compared to complete genotypes

Factor/Assay (Condition)	Complete^a^			Partial Genotype^b^ Imbalances									No Genotype^c^ Imbalances								
	Total	Partial	None	Total			Known variants			Predicted variants			Total			Known variants			Predicted variants		
	N_t_	N_imp_	N_com_	N_t_	Sens	Prec	N_imp_	Sens	Prec	N_t_-N_imp_	Sens	Prec	N_t_	Sens	Prec	N_com_	Sens	Prec	N_t_-N_com_	Sens	Prec
CREB1 (50 bp)	200	125	141	190	73.0	76.8	134	96.8	90.3	56	33.3	44.6	203	76.0	74.9	160	93.6	82.5	43	33.9	46.5
CREB1 (35 bp)	106	70	81	104	73.6	75.0	74	97.1	91.9	30	27.8	33.3	107	77.4	76.6	87	92.6	86.2	20	28.0	35.0
CREB1 (20 bp)	26	16	16	24	69.2	75.0	17	100.0	94.1	7	20.0	28.6	22	69.2	81.8	17	100.0	94.1	5	20.0	40.0
CTCF (35 bp)	267	187	192	300	83.1	74.0	198	98.4	92.9	102	47.5	37.3	298	85.0	76.2	210	97.9	89.5	88	52.0	44.3
DNase (20 bp)	104	43	47	138	51.0	38.4	42	97.7	100.0	96	18.0	11.5	144	51.9	37.5	55	97.9	83.6	89	14.0	9.0
CREB1 (2 alns)^d^	200	125	141	195	78.5	80.5	135	97.6	90.4	60	46.7	58.3	204	77.0	75.5	156	92.2	83.3	48	40.7	50.0
Mismatches alllowed																					
CREB1 (0 mm)	199	122	138	137	58.8	85.4	137	95.9	85.4	0	-	-	160	63.3	78.8	160	91.3	78.8	0	-	-
CREB1 (1 m m)^e^	200	125	141	190	73.0	76.8	134	96.8	90.3	56	33.3	44.6	203	76.0	74.9	160	93.6	82.5	43	33.9	46.5
CREB1 (2 mm)	199	124	137	245	80.4	65.3	133	97.6	91.0	112	52.0	34.8	251	81.4	64.5	159	96.4	83.0	92	48.4	32.6
CREB1 (3 mm)	213	123	143	301	79.2	53.2	132	98.4	90.9	169	50.0	23.7	313	81.7	52.7	161	96.4	83.9	152	47.6	19.7
Minimum reads/allele																					
CREB1 (2 reads)	301	178	199	486	73.4	45.5	187	97.2	92.5	299	39.0	16.1	515	75.4	44.1	228	95.0	82.9	287	37.3	13.2
CREB1 (3 reads)	261	156	173	267	70.1	68.8	162	94.9	91.4	105	33.3	33.3	289	72.8	65.7	191	92.5	83.8	98	34.1	30.6
CREB1 (4 reads)	230	142	159	218	71.4	76.0	148	95.1	91.2	70	33.7	42.6	235	74.8	73.2	175	92.5	84.0	60	35.2	41.7
CREB1 (5 reads)^e^	200	125	141	190	73.0	76.8	134	96.8	90.3	56	33.3	44.6	203	76.0	74.9	160	93.6	82.5	43	33.9	46.5
CREB1 (6 reads)	198	122	136	174	70.7	80.5	130	96.7	90.8	44	28.9	50.0	188	73.7	77.7	153	93.4	83.0	35	30.6	54.3
CREB1 (7 reads)	173	109	123	154	72.8	81.8	116	97.2	91.4	38	31.2	52.6	167	75.7	78.4	138	92.7	82.6	29	34.0	58.6
CREB1 (8 reads)	157	100	111	141	72.0	80.1	107	97.0	90.7	34	28.1	47.1	148	75.2	79.7	124	92.8	83.1	24	32.6	62.5
CREB1 (9 reads)	144	91	101	130	72.2	80.0	98	96.7	89.8	32	30.2	50.0	140	75.7	77.9	115	93.1	81.7	25	34.9	60.0
CREB1 (10 reads)	124	80	88	117	74.2	78.6	88	96.2	87.5	29	34.1	51.7	125	76.6	76.0	102	92.0	79.4	23	38.9	60.9
CREB1 (15 reads)	88	60	66	82	77.3	82.9	66	96.7	87.9	16	35.7	62.5	88	80.7	80.7	76	92.4	80.3	12	45.5	83.3
CREB1 (20 reads)	63	47	52	64	84.1	82.8	53	97.9	86.8	11	43.8	63.6	67	88.9	83.6	60	96.2	83.3	7	54.5	85.7
Imputation Rsq threshold																					
CREB1 (Rsq > .3) ^e^	200	125	-	190	73.0	76.8	134	96.8	90.3	56	33.3	44.6	-	-	-	-	-	-	-	-	-
CREB1 (Rsq > .4)	200	122	-	190	72.5	76.3	133	97.5	89.5	57	33.3	45.6	-	-	-	-	-	-	-	-	-
CREB1 (Rsq > .5)	200	121	-	187	72.5	77.5	129	98.3	92.2	58	32.9	44.8	-	-	-	-	-	-	-	-	-
CREB1 (Rsq > .6)	200	118	-	186	72.5	78.0	124	98.3	93.5	62	35.4	46.8	-	-	-	-	-	-	-	-	-
CREB1 (Rsq > .7)	200	117	-	185	72.0	77.8	123	98.3	93.5	62	34.9	46.8	-	-	-	-	-	-	-	-	-
CREB1 (Rsq > .8)	200	104	-	182	70.5	77.5	111	99.0	92.8	71	39.6	53.5	-	-	-	-	-	-	-	-	-
CREB1 (Rsq > .9)	200	96	-	176	69.5	79.0	99	99.0	96.0	77	42.3	57.1	-	-	-	-	-	-	-	-	-

^a^Complete genotype alignments use sequencing-based genotypes ^b^Partial genotype alignments use array-based genotypes and imputation ^c^No genotypes alignments use common variants (MAF > .05) from 1000 Genomes EUR ^d^Imbalances called after a second alignment using refined genotypes; known variants are variants included in the first alignment ^e^Condition used by default by AA-ALIGNER; N_t_ total imbalance count, N_imp_ imbalances at heterozygous sites identified by imputation, N_com_ imbalances at common variants, Sens, percent sensitivity, Prec, percent precision

We considered whether poor performance at predicted heterozygous sites was due to either (i) incorrect identification of homozygous sites as heterozygous using sequencing data [38]; or (ii) incorrect classification of balanced heterozygous sites as imbalanced due to alignment biases. By comparing the complete genotypes from genomic sequencing to imbalances at sites predicted to be heterozygous in the sequence data, we found that of the sites incorrectly predicted to be imbalanced, 58 % (18 of 31) using partial genotypes and 83 % (19 of 23) using common variants were not heterozygous. When using complete genotype information, AA-ALIGNER does not report imbalances at predicted heterozygous sites. Of the imbalanced sites, 61 % (11/18) using partial genotypes and 42 % (8/19) using common variants were also imbalanced when using complete genotypes, underscoring the difficulty in using short reads to detect imbalances at predicted heterozygous sites. We incorrectly detected imbalance at 13 sites using partial genotypes and 4 sites using common variants because an increase or decrease in aligned reads containing one allele now caused the site to pass the significance threshold for imbalance.

We tested whether a more stringent binomial *p*-value threshold than 0.01 would improve performance, by reducing errors resulting from condition (ii). As expected, a stricter threshold reduced the number of imbalances detected, but it also decreased sensitivity and precision (Additional file 2: Table S2), especially at predicted heterozygous sites. Additionally, we found at predicted heterozygous sites the *p*-values of false positive imbalance sites were more significant than the *p*-values of true positives sites when using partial genotypes (Mann–Whitney U *P* = .003) and common variants (Mann–Whitney U *P* = .03; Additional file 1: Figure S2)**.** These data suggest that errors in imbalance detection result more commonly from incorrect prediction of heterozygous sites than falsely calling imbalances at true heterozygous sites.

In addition to a binomial test, other statistical methods of detecting allelic imbalance have been used to measure the significance of allelic imbalance [21, 22, 25]. For example, a beta-binomial test is commonly used to correct for inaccurate imbalance detection caused by over dispersion of the data. Using a beta-binomial test (*P* < .01) for the 50 bp pair CREB1 ChIP-seq data reduced the number of sites of allelic imbalance identified by 82-83 % using complete, partial or no genotype information (Additional file 2: Table S2). Overall sensitivity and precision of imbalance detection using partial or no genotypes declined to ~50 %. Sensitivity and precision remained higher at imputed heterozygous sites (partial genotype alignment) and common variants (no genotype alignment) than predicted and uncommon variants as before. This reduction in the sensitivity and precision of imbalance detection is similar to the reduction seen when using a stricter binomial *p*-value threshold and is likely related to the increased *p*-values of false positive sites reported above.

We also considered whether common variants could be annotated more accurately than rare variants due simply to how sequences were aligned to these sites. Using BWA alignments that did not include any variant information, we predicted heterozygous sites and allelic imbalances as above. If we separate these predictions into those sites that are and are not common variants, we find that the sensitivity and precision are significantly higher for common variants (Additional file 2: Table S1**)**, although still lower than when both alleles were included in the alignment.

### Second alignment provides only modest improvement in sensitivity and precision for incomplete genotypes

Previously, Ni et al. [20] described a strategy for detecting allelic imbalance that first identifies heterozygous sites using an initial alignment without variant information, and then performs a second, allele-aware alignment including the predicted variants. We tested whether a similar second alignment would boost the sensitivity and precision of allelic imbalance identification at predicted heterozygous sites. Before the second alignment, the customized reference was updated to ensure that one allele was present at each heterozygous site predicted in the initial alignment, and non-reference alleles were added to the separate variant file. Reads were then re-aligned using this updated variant file and reference, and filtered as before.

Considering the CREB1 data with partial genotype information, this second alignment identified 11 additional correct sites of allelic imbalance while eliminating 6 incorrect sites, increasing the sensitivity to 47 % and precision to 58 % at predicted heterozygous sites (Table 2). When using common alleles, two additional correct imbalances were found and one incorrect site eliminated, with little change in sensitivity and precision. While a second, allele-aware alignment increases accuracy at predicted heterozygous sites, these modest gains, still accompanied by a high rate of false discovery, require an additional alignment. For all other analyses, we report imbalances detected after a single alignment.

### Shorter read length and lower sequencing depth reduce the number of imbalance predictions but not precision or sensitivity

Most existing ChIP-seq datasets, such as from ENCODE, contain sequence reads shorter than 50 bp. We investigated how read length affects the ability of AA-ALIGNER to identify sites of allelic imbalance by trimming the 3′ end of each 50 bp CREB1 ChIP-seq sequence to create 35 bp and 20 bp reads and then aligned these as before. Trimming reduced the overall number of sequenced bases considered by 30 % and 60 %, respectively. The total number of aligned reads decreased by 3.7 % in the 35 bp alignment and 16.7 % in the 20 bp alignment, further reducing total base coverage. The number of reads overlapping heterozygous sites decreased by 31.3 % and 61.9 %, respectively (Additional file 1: Figure S3A), which led to an even greater reduction in number of identified allelic imbalances for 35 bp (106 imbalances; 47.0 % reduction) and 20 bp (26 imbalances; 86.6 % reduction) reads (Table 2, Additional file 1: Figure S3B).

To determine whether reduced allelic imbalance detection was simply due to lower overall base coverage, we randomly sampled 70 % and 40 % of the 50 bp reads to match total base coverage levels for the above experiments using 35 bp and 20 bp reads. We found that the number of reads aligned to heterozygous sites decreased, as did imbalances identified, at the same rate as with the shorter reads (Additional file 1: Figure S3C). Thus, reducing base coverage had a proportionate effect on allelic imbalance identification compared to reduction in mapping to heterozygous sites. In our original analysis using all 50 bp reads, we noted 22.5 % of sites passed the threshold for the minimum number of reads required for each allele to be tested for imbalance by three reads or less (Additional file 1: Figure S3D). As base coverage is reduced, a disproportionate number of these sites then fall below that threshold (N = 5).

As expected, the overall number of predicted imbalance sites also decreased with base coverage when using complete genotypes. Compared to the imbalances detected with complete genotypes for each read length, the sensitivity of imbalance calls using partial genotypes or common variants remained greater than 69 % and the precision greater than 75 %. These data demonstrate that AA-ALIGNER maintains high detection accuracy using partial genotypes or common variants compared to complete genotypes with reduced base coverage.

### Number of imbalances identified varies across factors and assays

To ensure that the results from the CREB1 dataset were representative of results from other experiments, we used AA-ALIGNER to predict allelic imbalance in twelve additional transcription factor ChIP-seq datasets and one DNase-seq dataset generated in the same GM12878 cell line. ChIP-seq datasets contained between 14 and 48 million aligned reads, and most reads were 36 bp in length. Overall, we found that for all alignments, imbalance predictions were accurately replicated using incomplete genotypes at sites where both alleles were used in the alignment. Imbalances at new heterozygous sites were again very poorly predicted (Additional file 2: Table S3).

Although the precision of imbalance detection using partial genotypes and common variants was high across datasets, the number of imbalances detected varied greatly (minimum = 0, maximum = 291, median 19). Read length and sequencing depth influenced the ability of AA-ALIGNER to identify sites of imbalance (Additional file 1: Figure S3). We found, though, that measurements related to these characteristics (Additional file 1: Figure S4A-C) were not highly correlated with the number of imbalances detected in these ChIP-seq datasets (0.43 ≥ Pearson R^2^ ≥ 0.51). These low correlations suggest that other factors, such as the number of transcription factor binding sites (TFBS) across the genome and their overall genomic coverage also influenced imbalance detection. Alone, TFBS genomic coverage (Additional file 1: Figure S4D) showed low correlation with the number of imbalances detected (Pearson R^2^ = .35), but measurements that considered sequencing depth, read length and genomic coverage together (Additional file 1: Figure S4E-G) were highly correlated with the number of imbalances detected (0.78 ≥ Pearson R^2^ ≥ 0.91). These correlations suggest that the dispersion of sequence signal across the genome needs to be considered in addition to read length and sequencing depth when evaluating the potential of AA-ALIGNER to identify allelic imbalances. While there was a positive correlation between sequencing depth and signal dispersion in ChIP-seq data, the DNase-seq data, had greater sequencing depth (aligned reads) and signal dispersion (genomic coverage), but fewer sites of allelic imbalance identified than some of the ChIP-seq data. These results suggest that sequencing depth and signal dispersion influence imbalance in DNase-seq data differently and that the correlations observed in the ChIP-seq data do not extend to DNase-seq (Additional file 2: Table S4).

### Allowing additional alignment mismatches increases sensitivity but decreases precision

Parameters for the different steps of allelic imbalance identification vary across reported methods and can significantly affect results. Increasing allowed alignment mismatches helps overcome missing genotypes, inaccuracies in the reference genome, and errors in the sequence reads, but also results in increased erroneous sequence alignment, particularly when aligning shorter reads. We examined how this parameter affected the performance of AA-ALIGNER with limited genotype information. The 50 bp CREB1 data was processed with complete genotypes, partial genotypes, and common variant information allowing 0, 1, 2 or 3 alignment mismatches. With complete genotype information, the number of imbalances increased only slightly with greater mismatches (<4 %; Table 2).

When using partial genotypes or common variants, aligning with zero mismatches reduced the number of incorrectly aligned reads compared with our default of one mismatch, but at the cost of eliminating reads containing the non-reference allele at heterozygous sites not included during alignment. This led to increased overall precision of imbalance identification, but with significant loss of sensitivity as novel variants could not be predicted (Table 2). Of note, the precision of imbalance detection at known variants using zero mismatches was lower than when allowing one mismatch. Allowing two or three mismatches increased the number of imbalance sites identified using incomplete genotypes by more than 29 % (Table 2). The precision at variants included in the alignment did not change, but was greatly reduced at predicted variants, indicating less stringent mismatch thresholds increase the number of misaligned reads resulting in spurious predictions of heterozygous sites and allelic imbalance at these sites. We also tested whether requiring one of the mismatches to be located at the predicted heterozygous site increased sensitivity and precision compared to allowing mismatches at any site and found that results were similar in both cases (data not shown).

### Requiring a minimum number of reads containing each allele increases precision at predicted heterozygous sites

To balance sensitivity and precision with incomplete genotype information, we examined the impact of changing the minimum aligned read threshold for each allele required to test for imbalanced sites. Using the 50 bp CREB1 data, we found that as the required number of aligned reads increased from 2 to 10, the number of detected imbalances decreased using any level of genotype information, as expected, with small fluctuations in the overall sensitivity of imbalance identification using incomplete genotypes (Table 2). At thresholds of 15 and 20 reads per allele, the sensitivity of detection increased at predicted heterozygous sites, boosting the overall sensitivity at these thresholds. When considering imbalances at variants with both alleles included in the alignment, precision only varied slightly, but it increased at predicted sites with higher thresholds. While for most analyses we have required at least five reads per allele, these findings suggests that for known heterozygous sites, using a lower threshold will increase the number of identified sites without compromising precision.

### Requiring higher imputation quality does not significantly improve imbalance identification

For each variant on the genotyping array, imputation quality (Rsq) reflects confidence in imputation of that variant within the population of genotyped individuals. As the imputation quality of a variant site increases, our confidence in the accuracy of the genotype assigned in GM12878 also increases. Poorly-imputed variants incorrectly identified as heterozygous in GM12878 and included during alignment can lower the precision of imbalance detection using partial genotype information. Using imputation quality thresholds from 0.3 to 0.9 as a requirement of inclusion during alignment, we tested the influence of stricter thresholds on imbalance precision and sensitivity using partial genotypes. When using a higher threshold of 0.9, some variants with a quality between 0.3 and 0.9 were still predicted to be heterozygous, increasing the precision of imbalance detection at predicted sites, but overall using a threshold of 0.9 reduced the number of false positive sites by 7 compared to 0.3 while decreasing the number of true positive sites by the same amount, resulting in a small increase in precision and decrease in sensitivity (Table 2).

### Allelic differences in CREB1 binding experimentally supported at inflammatory bowel disease-associated loci and other predicted sites

**Fig. 2:**
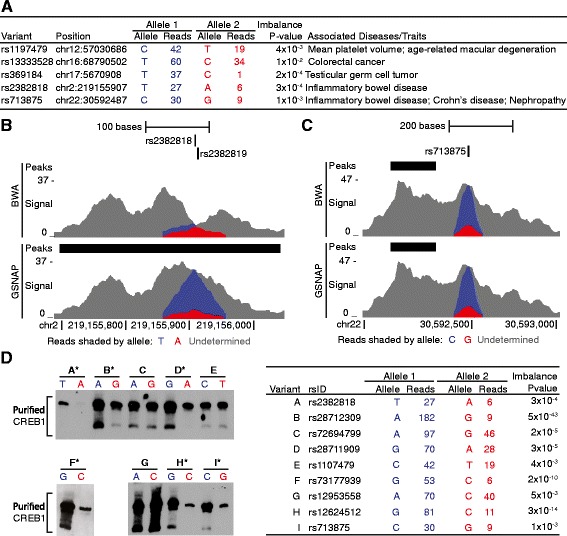
Validation of allelic imbalance detected at GWAS loci and other predicted sites. **a** We detected significant allelic imbalance (binomial *P* < 0.01) in CREB1 ChIP-seq sequence reads at variants at five disease- and trait-associated loci. **b** At rs2382818, sequence reads that failed to align when only single alleles were considered (top) were correctly aligned in an allele-aware alignment (bottom). The increase in aligned reads allowed for the detection of a CREB1 peak (black box) and allelic imbalance at the variant for which more reads were aligned containing the T allele than the A allele were aligned. Total sequence signal is displayed and reads are shaded based which allele they contain. **c** We detected a significantly greater proportion of reads containing the C allele of rs713875 than the G allele. **d** EMSA using purified CREB1 and labeled probes containing each allele at nine sites of allelic imbalance to test for allelic differences in binding. Alleles colored blue are predicted to bind CREB1 more strongly than alleles colored red. Allelic differences in protein binding consistent with these predictions were observed for starred (*) variants. Only CREB1-bound probe is shown. Similar results were observed in a replicate experiment

The above analyses assume that imbalances detected using complete genotypes are the most accurate for comparing the effects of reduced information and parameter settings, but they do not address the functional accuracy of the imbalance prediction. Of special interest are sites previously shown to be associated with disease, especially a disease for which the GM12878 lymphoblastoid cell line is relevant. We identified 238 heterozygous sites in GM12878 that are in linkage disequilibrium (1000 Genomes EUR; r^2^ ≥ .8) with one of 218 index SNPs reported for a genome wide association study (GWAS, *P* < 1.0x10^−5^) [39]. AA-ALIGNER predicted allelic imbalances (*P* < 0.01) in CREB1 binding in GM12878 at five of these disease-associated loci (Fig. 2a). Two of the sites, rs2382818 (Fig. 2b) and rs713875 (Fig. 2c), are at loci associated with inflammatory bowel disease susceptibility [40–42]. CREB family proteins have previously reported links to inflammation [43], B-cell lymphocytes [44], and inflammatory bowel disease [44].

At rs2382818, 27 reads containing the T allele and 6 reads containing the A allele were aligned using complete genotype, partial genotype, and common variant information (binomial *P* = 3.2x10^−4^; Fig. 2b, bottom panel). The T allele of rs2382818 most often segregates with the disease risk allele A of rs2382817 [44]. Electrophoretic mobility shift assays (EMSAs) using purified CREB1, conducted in the absence of chromatin and other nuclear proteins, can experimentally test for differential binding of CREB1 to a specific DNA sequence. Multiple, independently performed EMSAs supported allelic differences in binding at rs2382818 (Fig. 2d). A second heterozygous site is located 2 bp downstream of rs2382818. Allowing only a single mismatch during alignment prevents reads from aligning if both alleles are not considered. At this site, a peak and an allelic imbalance were only detected when using GSNAP alignments, but not BWA (Fig. 2b), demonstrating the importance of using allele-aware alignments in annotating disease-associated variants. This locus has been annotated as an enhancer based on ENCODE histone modification data [45] and linked with the expression of nearby genes (*SLC11A1, USP37, PNKD*, and *ZNF142*) [46]. We used MEME-ChIP [47] to identify a CREB1 binding motif from the 10,000 strongest ChIP-seq peaks and searched for the presence of this motif at rs2382818 using FIMO (e < 1.0x10^−5^) [48], but we were unable to detect the CREB1 motif at this site.

At rs713875 (*MTMR3* locus), 30 reads containing the Crohn’s disease risk C allele [42] and 9 reads containing the G allele were aligned using any level of genotype information (binomial *P* = 1.1x10^−3^; Fig. 2c). Allelic differences in CREB1 binding were again supported by EMSA (Fig. 2d). In this example, the imbalance was detected even when only one allele was used in the alignment. Rs713875 is contained within a DNaseI hypersensitive site and is predicted to function as an enhancer [45]. Correlation between DNaseI hypersensitivity and gene expression levels suggests that this locus may regulate nearby genes *LIF* and *TBC1D10A*, pseudogene *CTA-85E5.7*, and non-coding RNA *RP3-438O4.4* [49]. Of these, leukemia inhibitory factor (*LIF*) is an IL-6 cytokine believed to have both inflammatory and anti-inflammatory roles [50]. As with rs2382818, we were unable to detect a CREB1 binding motif at this site. For both rs713875 and rs2382818, further study would be required to show whether allelic differences in CREB1 binding alter transcription and affect inflammatory bowel disease.

We tested for allelic differences in CREB1 binding at seven additional sites that contain a CREB1 binding motif and were predicted to be imbalanced by AA-ALIGNER. These seven included rs1107479, which has been associated with mean platelet volume [51] and age-related macular degeneration [52]. Using EMSA, we detected evidence of allelic differences in protein binding in the same direction as our predicted imbalance at 4 of the 7 sites (Fig. 2d), for a total of 6 of 9 supported imbalances. Surprisingly, at rs1695359, we consistently detected increased protein binding for the allele predicted by our imbalance analysis to have decreased binding. Of the 6 EMSA-supported sites, only 3 were predicted to have allelic differences based on the FIMO-calculated motif score (difference > 5). Of the 3 imbalance sites that were not supported by EMSA, only one (rs1695359) had a significant difference in motif binding score, and the allele with the stronger motif score demonstrated increased binding in the EMSA result, rather than the allele predicted to be enriched by imbalance detection. For comparison, we used EMSA to test 5 additional CREB1 binding locations with a heterozygous variant that fell within a CREB1 binding motif, but were not predicted as sites of allelic imbalance (*P* > .3). We found evidence of allelic differences in protein binding at two of these sites (Additional file 1: Figure S5). For these two sites, a CREB1 motif was only predicted when the allele with stronger protein binding was present.

These data provide strong supporting evidence of allelic differences in protein binding at 6 of the 9 predicted imbalanced sites and suggest that the sequence-specific binding preferences of CREB1 influence binding at these sites. It is unclear whether the remaining three sites not supported by EMSA indicate errors in AA-ALIGNER imbalance detection, or whether these show limitations of EMSA in detecting *in vivo* allelic differences in protein binding that are dependent on chromatin context or the presence of other nuclear proteins. Likewise, it is unclear whether AA-ALIGNER failed to detect allelic imbalance at two sites with allelic differences in protein binding based on EMSA, or whether chromatin and/or other proteins compensate for reduced sequence specificity *in vivo* resulting in similar binding regardless of allele present. Overall, these EMSA results provide evidence supporting allelic differences in protein binding at individual imbalance sites detected by AA-ALIGNER.

## Discussion

In this study, we have demonstrated the ability of AA-ALIGNER to remove mapping biases and to identify allelic imbalance with high sensitivity and precision when using partial or no prior genotype information compared to using complete genotype information. Thoroughly testing allelic imbalance detection using three levels of genotype information provides a clear picture of the accuracy of AA-ALIGNER when using limited genotypes compared to complete genotypes.

This is the first in-depth study of allelic imbalance detection in ChIP-seq and DNase-seq data that empirically tested the effects of key aspects of these analyses including genotype availability, read length, alignment parameters, imputation parameters, and requirements for predicting heterozygous sites. Our results indicate that including any amount of genotype information, or both alleles at common variants, significantly increases accuracy of imbalance detection compared to predictions when complete genotypes are known. We clearly show that predicting heterozygous variants with these short read data is highly inaccurate, leading to false positive rates of imbalance detection greater than 50 %. We used a simple metric to predict heterozygous sites, and so one could argue that more sophisticated prediction methods could improve performance. A recent study examining the accuracy of genotyping with short reads from genomic sequencing found that removing sites with strong allelic imbalance, the very sites we are trying to identify, increased genotype accuracy [30]. That study highlighted the difficulty of identifying heterozygous sites from ChIP-seq and DNase-seq data, especially at imbalanced sites. Taken together with our data we strongly suggest that predicted genotypes should be further validated before embarking on functional analyses.

Predicting heterozygous sites in genome sequencing data is an active area of research, and many studies have demonstrated the difficulty of calling variants in sequencing data [25, 30, 53]. In addition to the GM12878 genotype annotation used in this study, other generally more conservative annotations exist. We found that most predicted imbalances were at common variants, and even when all common variants were included in alignments in the case of no genotypes, the true heterozygous variants and imbalances could be predicted well at these common variant sites. In contrast, the accuracy of imbalance detection at predicted heterozygous sites corresponding to rare variants is poor, even when these predicted heterozygous sites were included in a second alignment. Inaccurate imbalance detection can be caused by either i) incorrectly predicted heterozygous sites in the sequencing data (false positives) or ii) correctly predicted heterozygous sites in the sequencing data that were incorrectly annotated in the complete genotype (false negatives). Requiring more evidence to predict heterozygous sites increased the accuracy of imbalance detection, suggesting that false positives in heterozygous site predictions contributed to inaccurate imbalance detection. These incorrect predictions may be partly due to sequencing errors, but as some are still present at high minimum read thresholds, errors in sequence mapping likely contribute to false positives. The inclusion of incorrectly annotated heterozygous sites or absence of true heterozygous sites during sequence alignment can cause erroneous read mappings to highly similar genomic regions leading to incorrect heterozygous site identification.

Interestingly, many imbalances at sites not annotated as heterozygous in the complete genotype would have been considered imbalanced in the complete genotype alignment using our criteria. This suggests that errors may exist in the complete genotype data leading to false negative imbalance predictions. Further study is needed, but these data suggest that both false positives and false negatives contribute to decreased detection accuracy at predicted variants. Thus, AA-ALIGNER outputs three sets of detected imbalance sites: i) a complete set of all imbalances identified; ii) imbalances at known or common heterozygous variants (higher confidence); and iii) imbalances at predicted rare variants (lower confidence).

We showed that simply including both alleles for common variants resulted in annotations nearly as accurate as those generated from imputed genotypes. Including information about rare variants may further increase sensitivity of imbalance detection. We only considered imputed genotypes and common variants separately, but carefully combining information from these sources may perform better than either individually and is an area of future research.

Other tested parameters demonstrated the trade-off between sensitivity and precision based on their settings, but in most cases these parameters had little effect other than to change the number of predicted imbalanced sites. Nevertheless, these results can be used to guide the analysis of new data, and AA-ALIGNER allows for the easy specification of these parameters. For example, it may be prudent to apply different criteria when evaluating variant sites with known genotypes or that are common variants compared to those predicted to be heterozygous based solely on the short read data. For most of our results, we required a minimum of five reads per allele when testing for imbalances to prevent erroneous testing of homozygous variants. When strong evidence exists for heterozygosity, though, this requirement may be loosened or eliminated, allowing for greater sensitivity in identifying more extreme imbalances. While it is prudent to require a minimum read threshold of reads to detect imbalances at predicted heterozygous sites, this threshold precludes the identification of complete imbalance at known heterozygous sites where only one allele is present, such as imprinted loci. When using known heterozygous sites, AA-ALIGNER users have the option to detect complete imbalance at these sites.

The lack of a comprehensive catalog of experimentally validated sites with functional allelic differences limits our ability to evaluate allelic imbalance predictions. Our study used results obtained from complete genotypes, the best-case scenario for imbalance detection, as the standard for evaluating analyses with partial genotypes and common variants. We experimentally tested for allelic differences in CREB1 binding using EMSA at nine sites with predicted allelic imbalance and five sites with no predicted imbalance. In general, EMSA results matched predicted differences in FIMO-calculated motif scores based on the presence of each of the two alleles, though we note that we were able to detect allelic imbalance and observe differential protein binding at three sites without predicted allelic differences in motif scores. EMSAs were performed in the absence of chromatin context and other nuclear proteins, and so are limited to detecting differences in the sequence binding specificity of a protein. Despite this limitation, we detected allelic differences in CREB1 binding at 6 of 9 predicted imbalanced sites providing strong supporting evidence of allelic differences in protein binding. Further testing is required to understand the cases when EMSA results do not support predicted allelic imbalances. For example, it is unknown whether any of the 3 sites not supported by EMSA were falsely detected as imbalanced by AA-ALIGNER, or whether they failed to validate because of the limitations inherent to EMSA. Likewise, further study is needed to determine whether the two sites that AA-ALIGNER did not predict as imbalanced but that EMSA showed allelic differences in protein binding are due to limitations in AA-ALIGNER or EMSA. These results highlight the need for better experimental assays to validate allelic imbalances, and underscore the difficulty of creating comprehensive catalogs of sites with experimental evidence of differences in protein binding.

The most appropriate statistical test and significance threshold for determining imbalanced sites is not known. While the binomial test is commonly used, other statistical methods such as a beta-binomial [21, 54], and Bayesian frameworks [25, 54] have been shown to accurately detect allelic imbalance. For our analyses, we used the more optimistic binomial test and determined significance using an uncorrected *p*-value threshold of 0.01. Our data indicate that stricter *p*-value thresholds do not significantly affect the sensitivity and precision of predictions using incomplete genotypes when compared to complete genotype annotations. Incorrectly predicted heterozygous sites often had very small *p*-values (25 % at *P* < 10^−7^), thus stricter *p*-values will not eliminate these false positives. Likewise, using beta-binomial *p*-values to correct for over dispersion and setting the same uncorrected *p*-value cut-off greatly reduced our power to detect allelic imbalance. Using the beta-binomial *p*-value, imbalance detection accuracy and precision remain significantly higher for imputed and common variants than for predicted rare variants. Our experimental EMSA results were strongest overall for sites with lower *p*-values, although we did show evidence for altered binding at rs713875 (binomial *P =* 1.0x10^−3^) and rs2382818 (*P* =3.2x10^−4^) but not rs72694799 (*P* =2.6x10^−5^) (Fig. 2d). Sites with less statistically significant changes in allelic data may be biologically inconsequential, or the functional effects may simply be weaker but still biologically significant. Until a larger set of experimentally supported sites exists, we cannot determine which statistical test and *p*-value threshold best identifies biologically relevant imbalance sites. AA-ALIGNER was designed to be modular allowing for allowing for the incorporation of alternative methods for variant identification and tests for significance of imbalances.

Copy number variants (CNVs), which can have significant impacts on disease [55], can cause one allele to overrepresented in the genomic DNA leading to biologically inconsequential imbalances in read data. Prior CNV information for the sequenced sample can be used to preclude imbalance detection within CNVs. Alternatively, sequence data from non-ChIP genomic input or other control experiments, when sequenced with sufficient read depth in the same sample, could be used to estimate an expected proportion of aligned reads per allele and to adjust for copy number variation within the binomial test. These control sequences could also correct for other biases that cause incorrect allelic imbalance detection in both the control and ChIP-seq data. Like genotype information, CNV data is not available for most samples. At this time, AA-ALIGNER does not specifically incorporate known CNV data, although known CNVs can easily be included as “blacklisted” regions and filtered post-alignment. Alternatively, presence of CNVs could be experimentally tested for at AA-ALIGNER predicted imbalance sites.

## Conclusions

Allelic imbalance analyses in quantitative sequence data from functional genomic experiments such as ChIP-seq and DNase-seq data is a powerful way to identify effects of genetic variation on gene regulation and uncover molecular mechanisms responsible for GWAS loci in non-coding genomic regions. Reference mapping biases at heterozygous sites and a lack of genotype information for sequenced samples greatly hinder allelic imbalance detection in most public *-seq data. Our analyses demonstrate that the AA-ALIGNER pipeline overcomes mapping biases and accurately identifies a majority of imbalance sites using only partial or no genotype information compared to complete genotype information. Additionally, we provide valuable insight into how experimental and methodological design factors effect imbalance detection.

With AA-ALIGNER, we were able to detect allelic imbalance in ChIP-seq data for a single transcription factor from a single cell line and provide supporting experimental evidence of differential protein binding at a small subset of imbalanced sites. These sites with experimental evidence included variants at two inflammatory bowel disease-associated loci. We demonstrated that mapping biases at one of these two sites prevented detection of both signal enrichment and allelic imbalance using standard analytical techniques. Existing knowledge of B-lymphocytes, regulatory regions and nearby genes suggest a plausible role for these imbalanced sites in inflammatory bowel disease pathogenesis, highlighting the utility of imbalance detection in annotating disease-associated loci. Replicating this analysis in additional cell lines and for additional factors should continue to uncover allelic imbalance at numerous other GWAS loci, providing powerful insight into likely genetic effects on regulation.

## Additional files

Additional file 1: 
**Supplemental Materials. Figure S1:** Reference mapping biases influence sequence alignment. Example of effect of reference mapping biases on sequence alignment. **Figure S2:** False positive imbalance sites have more significant p-values. P-values of imbalances detected, grouped by genotype availability and accuracy. **Figure S3:** Read length influences sequence alignment and allelic imbalance detection at heterozygous sites. (A) Alignment statistics of different reads with different lengths. Effects of sequence length (B) and depth (C) on imbalance detection. (D) Distribution of  number of reads for underrepresented allele. **Figure S4:** Correlation of alignment statistics and number of imbalances detected. **Figure S5:** Allelic differences in binding at sites without predicted allelic imbalance. EMSA results for sites with reads mapping but no detected imbalance. Additional file 2: 
**Supplemental Tables. Table S1:** Precision of imbalance detection in non-allele-aware alignments. Imbalance detection sensitivity and accuracy using BWA and no genotypes compared to GSNAP and common variants. **Table S2:** P-value threshold influences allelic imbalance detection using partial genotypes and common variants. Imbalance detection statistics using the binomial test and multiple thresholds or the beta-binomial test. **Table S3:** Precision of allelic imbalance detection extends to other transcription factor ChIP-seq data. Imbalance detection statistics for alignments of sequence data 11 additional proteins. **Table S4:** Alignment and base coverage statistics for ChIP-seq transcription factor and DNase-seq data. Alignment statistics for all alignments used in these analyses. **Table S5:** EMSA probes for experimental validation. **Table S6:** Heterozygous sites tested for allelic imbalance in complete genotype alignment (CREB1 50-bp). **Table S7:** Heterozygous sites tested for allelic imbalance in partial genotype alignment (CREB1 50-bp). **Table S8:** Heterozygous sites tested for allelic imbalance in no genotype alignment (CREB1 50-bp). 

## Abbreviations

ENCODE: Encyclopedia of DNA elements
bp: Base pairs
MAF: Minor allele frequency
TP: True positive
FP: False positive
FN: False negative
TFBS: Transcription factor binding site
GWAS: Genome wide association study
Rsq: Imputation quality
EMSA: Electrophoretic mobility shift assay
CNV: Copy number variants
