# Supplementary material for: Removing reference mapping biases using limited or no genotype data identifies allelic differences in protein binding at disease-associated loci
Source: BMC Med Genomics. 2015 Jul 26;8:43. doi: 10.1186/s12920-015-0117-x (PMC4515314; doi:10.1186/s12920-015-0117-x)
Supplement: Additional file 1: — Supplemental Materials. Figure S1: Reference mapping biases influence sequence alignment. Example of effect of reference mapping biases on sequence alignment. Figure S2: False positive imbalance sites have more significant p-values. P-values of imbalances detected, grouped by genotype availability and accuracy. Figure S3: Read length influences sequence alignment and allelic imbalance detection at heterozygous sites. (A) Alignment statistics of different reads with different lengths. Effects of sequence length (B) and depth (C) on imbalance detection. (D) Distribution of number of reads for underrepresented allele. Figure S4: Correlation of alignment statistics and number of imbalances detected. Figure S5: Allelic differences in binding at sites without predicted allelic imbalance. EMSA results for sites with reads mapping but no detected imbalance. [file 12920_2015_117_MOESM1_ESM.pdf]

Supplemental Materials

Removing reference mapping biases using limited or no genotype data identifies allelic differences in protein binding at disease-associated loci

Martin L. Buchkovich, Karl Eklund, Qing Duan, Yun Li, Karen L. Mohlke and Terrence S. Furey

Supplemental Figure Legends

Figure S1. Reference mapping biases influence sequence alignment. When mapping reads to a reference genome containing a single allele at heterozygous sites (left), sequence reads containing the reference allele (C, blue) are more likely to map correctly than reads containing the non-reference allele (A, red), especially in the presence of sequencing errors (orange). Reads containing each allele are equally as likely to map correctly when mapping reads to sequence containing both alleles at heterozygous sites (right).

Figure S2. False positive imbalance sites have more significant p-values. Boxplot of *P*-values at sites of allelic imbalance using complete (left), and partial (middle) genotypes and common variants (right). For the complete genotype alignment, *P*-values are further subdivided by inclusion in partial genotypes and common variants. For partial genotypes and common variant alignments, all *P*-values are displayed in addition to being divided into inclusion during alignment and predicted from the alignment, and then further divided into whether or not the sites was predicted using complete genotypes (false positive vs true positive). Subdivided groups with significant differences in *P*-value distributions (Mann Whitney U test; $P<.01$ and $P<.001$) are indicated.

Figure S3. Read length influences sequence alignment and allelic imbalance detection at heterozygous sites. (A) Alignment statistics for alignments of CREB1 ChIP-seq data, and when using different (B) read lengths and (C) sequence depths, plotted as percent of the 50 bp statistics. (D) Histogram of the number of reads containing the underrepresented allele at sites with significant imbalance (binomial $P<.01$, uncorrected) with 2 or more reads containing each allele. Vertical dashed line indicates the minimum of 5 or more reads containing each allele required for imbalance detection in (A).

Figure S4. Correlation of alignment statistics and number of imbalances detected. The number of sites of allelic imbalance in thirteen ChIP-seq and one DNase-seq dataset compared to the (A) total number of reads aligned; (B) number of reads aligned to heterozygous sites; (C) total number of bases aligned (read length x total reads aligned); the percent of genome with greater than (D) 1X and (E) 10X coverage; (F) the average read depth at bases with 1X or more coverage; (G) the ratio of sites with 10X coverage to 1X coverage and (H) the number of heterozygous sites identified. Pearson correlation R^2 values for each statistic and number of allelic imbalances are displayed for all data and only ChIP-seq data.

Figure S5. Allelic differences in binding at sites without predicted allelic imbalance. EMSA using purified CREB1 and labeled probes containing each allele at five sites with reads mapping but not significant allelic imbalance to test for allelic differences in binding. Alleles colored blue are the reference allele and alleles colored red are the other allele. Allelic differences in protein binding were detected at two sites (starred) without predicted imbalance but with predicted allelic differences in the presence of CREB1 motif. Only CREB1-bound probe is shown.

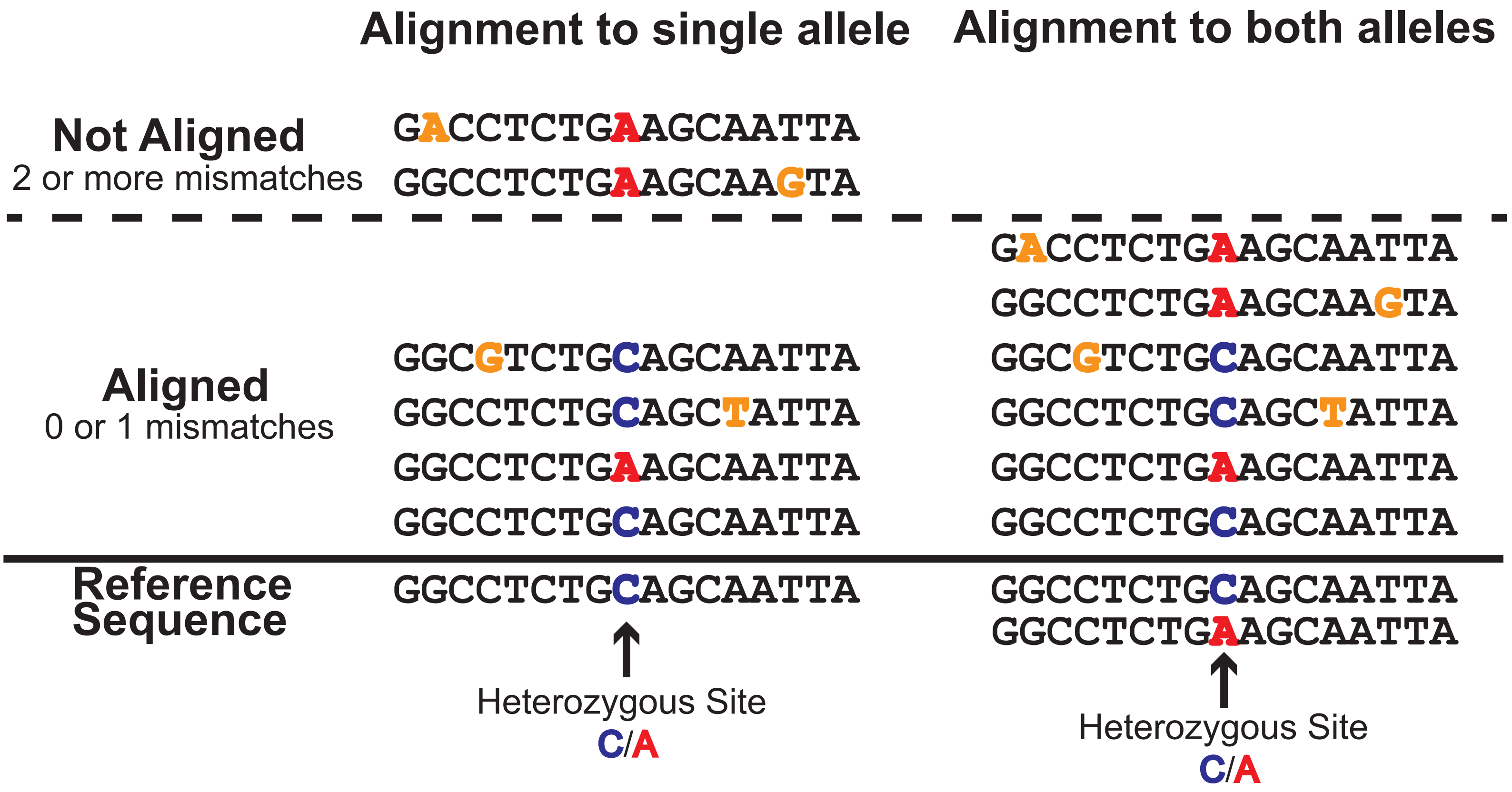
Alignment to single allele
Alignment to both alleles
Not Aligned
2 or more mismatches
GACCTCTGAAGCAATTA
GGCCTCTGAAGCAAGTA
GACCTCTGAAGCAATTA
GGCCTCTGAAGCAAGTA
Aligned
0 or 1 mismatches
GGCGTCTGCAGCAATTA
GGCCTCTGCAGCTATTA
GGCCTCTGAAGCAATTA
GGCCTCTGCAGCAATTA
GGCGTCTGCAGCAATTA
GGCCTCTGCAGCTATTA
GGCCTCTGAAGCAATTA
GGCCTCTGCAGCAATTA
Reference Sequence
GGCCTCTGCAGCAATTA
GGCCTCTGCAGCAATTA
GGCCTCTGAAGCAATTA
Heterozygous Site
C/A
Heterozygous Site
C/A

Figure S1

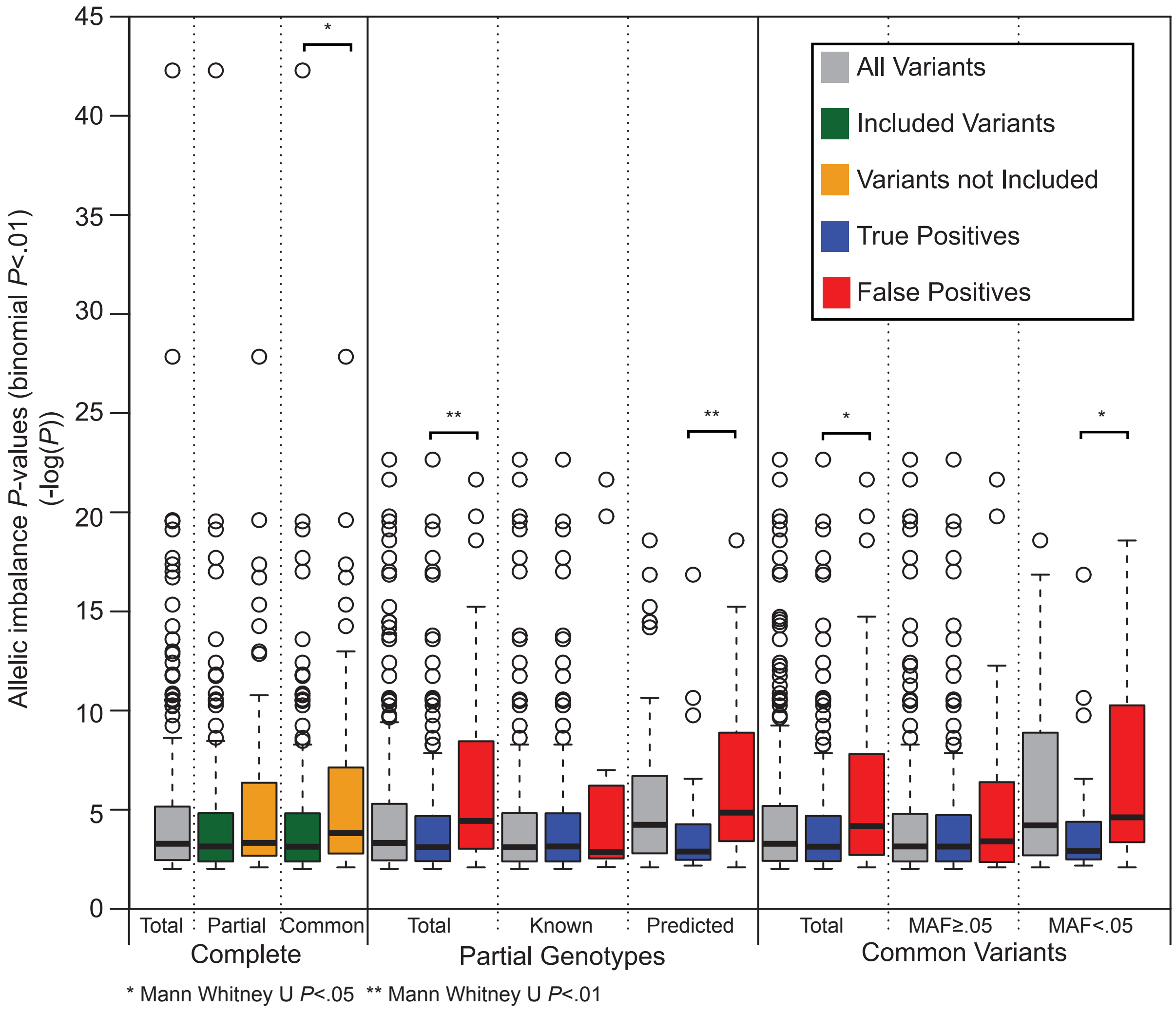

Allelic imbalance P-values (binomial P<.01) (-log(P))
45
40
35
30
25
20
15
10
5
0
All Variants
Included Variants
Variants not Included
True Positives
False Positives
*
**
**
*
*
Total
Partial
Common
Complete
Total
Known
Predicted
Partial Genotypes
Total
MAF≥.05
MAF<.05
Common Variants
* Mann Whitney U P<.05 ** Mann Whitney U P<.01

Figure S2

A

Factor	Condition	Total Reads Aligned	Reads at Heterozygous Sites	Allelic Imbalances
CREB1	50 bp	33,599,679	1,295,901	200
CREB1	35 bp	32,368,677	890,153	106
CREB1	20 bp	27,981,208	454,580	26
CREB1	Sampled 70%	23,518,477	907,345	123
CREB1	Sampled 40%	13,442,901	518,519	45

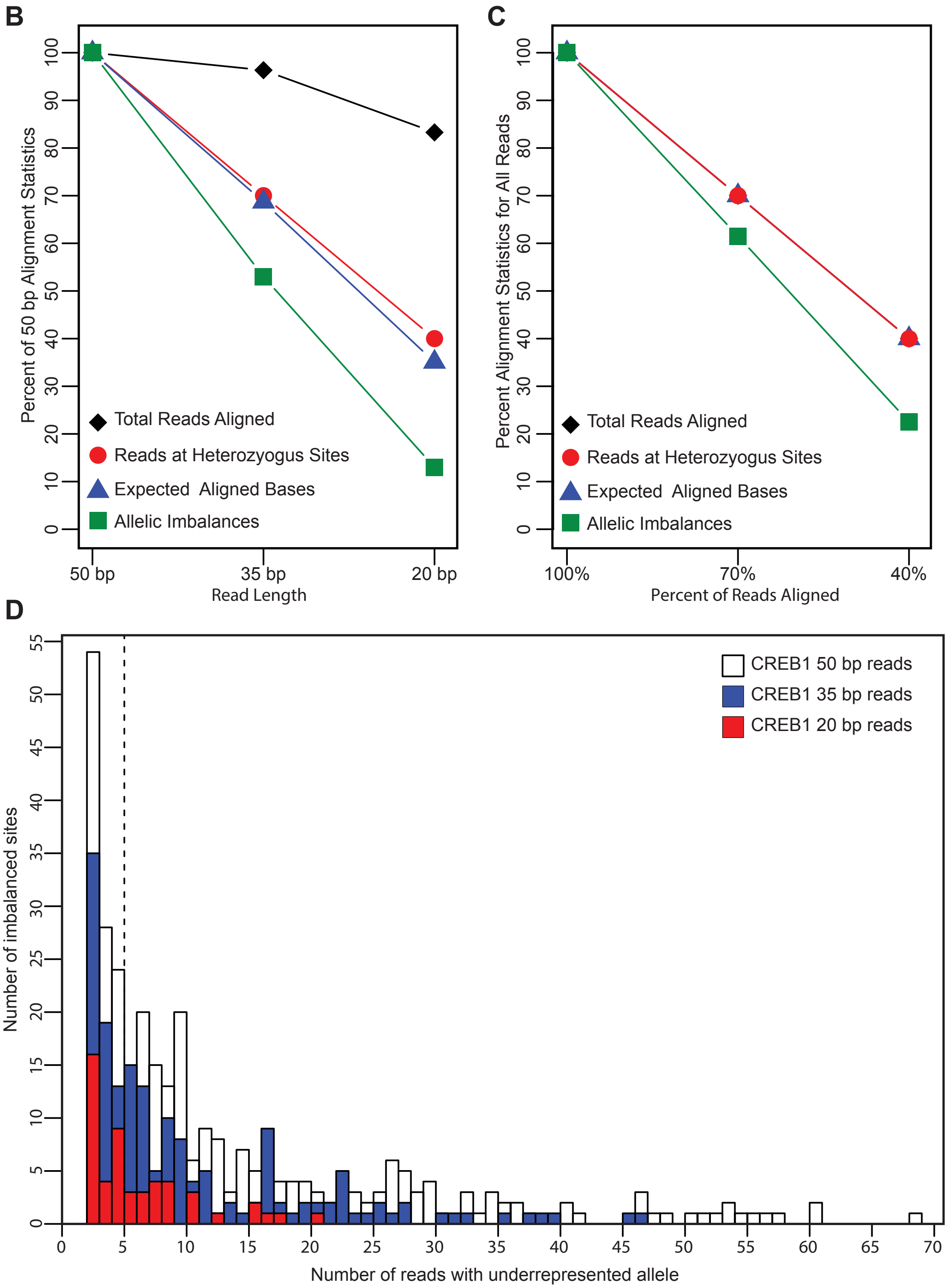

Figure S3

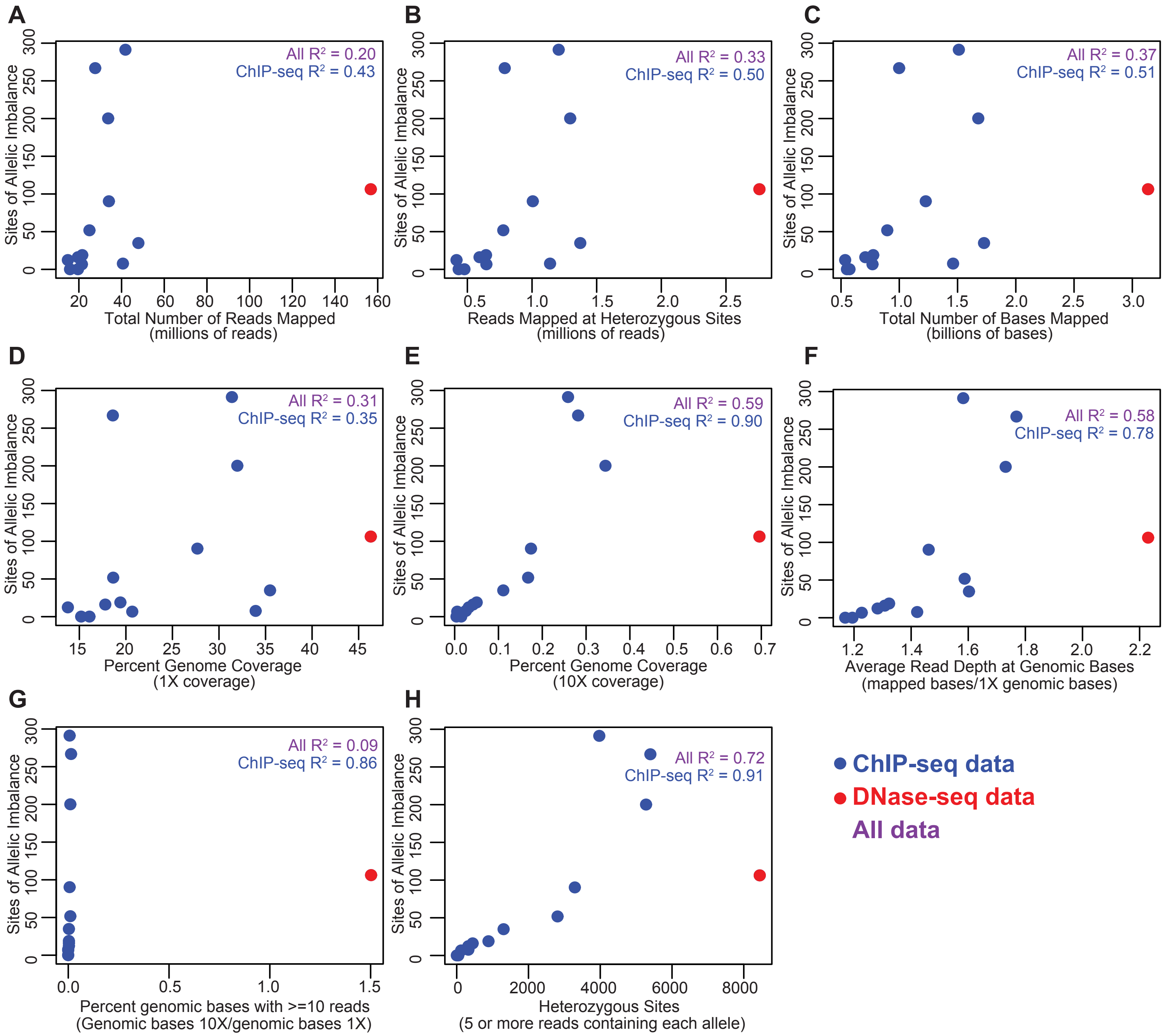
A
All R² = 0.20
ChIP-seq R² = 0.43
Sites of Allelic Imbalance
Total Number of Reads Mapped
(millions of reads)
B
All R² = 0.33
ChIP-seq R² = 0.50
Reads Mapped at Heterozygous Sites
(millions of reads)
C
All R² = 0.37
ChIP-seq R² = 0.51
Total Number of Bases Mapped
(billions of bases)
D
All R² = 0.31
ChIP-seq R² = 0.35
Percent Genome Coverage
(1X coverage)
E
All R² = 0.59
ChIP-seq R² = 0.90
Percent Genome Coverage
(10X coverage)
F
All R² = 0.58
ChIP-seq R² = 0.78
Average Read Depth at Genomic Bases
(mapped bases/1X genomic bases)
G
All R² = 0.09
ChIP-seq R² = 0.86
Percent genomic bases with >=10 reads
(Genomic bases 10X/genomic bases 1X)
H
All R² = 0.72
ChIP-seq R² = 0.91
Heterozygous Sites
(5 or more reads containing each allele)
ChIP-seq data
DNase-seq data
All data

Figure S4

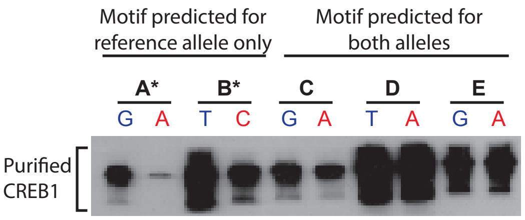

Variant	rsID	Allele 1		Allele 2		Imbalance
		Allele	Reads	Allele	Reads	P-value
A	rs72807213	G	24	A	17	0.35
B	rs9388486	T	36	C	33	0.81
C	rs274035	G	19	A	17	0.87
D	rs12145434	T	78	A	82	0.81
E	rs55811458	G	42	A	53	0.30

Table S5
